# Intracranial hemorrhage as the initial presentation of Sturge–Weber syndrome: a case report and literature review

**DOI:** 10.3389/fped.2026.1922107

**Published:** 2026-09-21

**Authors:** Yanmei Li, Jing Gan, Fan Ma, Fan Hu, Xiaoliang Liu

**Affiliations:** 1 Key Laboratory of Birth Defects and Related Diseases of Women and Children of MOE, Department of Pediatrics, West China Second University Hospital, Sichuan University, Chengdu, China; 2 West China Second University Hospital-Tianfu·Sichuan Provincial Children’s Hospital, Sichuan, China

**Keywords:** hemiparesis, intracranial hemorrhage, port-wine stain, seizure, Sturge–Weber syndrome

## Abstract

**Background:**

Sturge–Weber syndrome (SWS) is a rare congenital neurocutaneous disorder. Timely diagnosis and management remain challenging, particularly when intracranial hemorrhage (IH) occurs as a rare but serious complication.

**Case presentation:**

A young child developed IH after minor head trauma and presented with irritability, vomiting, gait instability, and lethargy. His symptoms progressed to recurrent focal motor seizures, status epilepticus, and hemiplegia. Initial CT showed a hematoma in the left frontal lobe, but SWS was not recognized immediately. Gadolinium-enhanced magnetic resonance imaging showed multiple gyriform enhancing linear shadows and leptomeningeal enhancement along the cortex extending into the sulci, consistent with pial angiomatosis. The diagnosis of SWS was established on the basis of the extensive port-wine stain (PWS), seizures, and neuroimaging findings. Intravenous midazolam relieved the seizures and restored consciousness, followed by sequential oral levetiracetam and valproic acid therapy. During follow-up, he remained seizure-free for more than two years while receiving anticonvulsants and low-dose aspirin, with only mild right hemiparesis.

**Conclusion:**

IH may be the first recognized neurological manifestation of previously undiagnosed SWS. In children with facial PWS and acute neurological symptoms, prompt neuroimaging may facilitate diagnosis and guide individualized management.

## 1 Introduction

Sturge–Weber syndrome (SWS) is a rare congenital neurocutaneous disorder with an estimated incidence of 1:20,000 to 1:50,000. It is characterized by vascular malformations involving the skin, eyes, and leptomeninges of the brain (1). A somatic R183Q mutation in the *GNAQ* gene has been identified as the main genetic alteration underlying SWS (2). Clinically, SWS may involve the skin, eyes, and brain, resulting in a wide spectrum of symptoms, comorbidities, disease severity, and outcomes. However, diagnosis is often delayed in clinical practice, partly because pediatricians may have limited awareness of the disorder (3). Some patients are not recognized until ocular or brain involvement becomes apparent, such as glaucoma or seizures (3, 4). Cho et al. reported that 16% of patients with SWS were diagnosed more than one year after the onset of initial symptoms, including birthmarks on the forehead and scalp, which are strongly associated with seizures (3). Patients with a large segmental port-wine stain (PWS) on the forehead are at particularly high risk of SWS (5).

Brain involvement in SWS most commonly presents with seizures, stroke-like episodes, hemiparesis, cerebral atrophy and calcifications, migraine headaches, intellectual disability, and visual field defects (1, 6). However, intracranial hemorrhage (IH) is a rare manifestation of SWS, with only nine cases reported to date (7–13). Among them, only four pediatric cases of SWS with IH have been described (7, 9, 11, 12). The intracranial venous malformations characteristic of SWS may increase the risk of hemorrhage, particularly after trauma (7). Early recognition and prompt intervention may therefore be critical for prognosis (8, 9). A more thorough understanding of brain involvement in SWS may also improve clinical management and outcomes. Here, we present the first pediatric case in China in which SWS presented with IH followed by seizures, with the aim of increasing awareness of this rare but important complication.

## 2 Case presentation

A 1-year-and-8-month-old boy presented to the emergency department with convulsions and lethargy. Four days before admission, he had fallen twice and sustained minor scalp abrasions with bleeding on the left forehead. The following day, he developed irritability, vomiting, and gait instability, followed by lethargy. Two days before admission, he developed recurrent right focal motor seizures that progressed to status epilepticus. He had no known exposure to suspected drugs or toxins. His birth history was unremarkable, and his developmental milestones were age appropriate. Before the current illness, he had no history of seizures, transient hemiparesis, staring spells, abnormal involuntary movements, recurrent unexplained vomiting, or other neurological or stroke-like episodes. There was no significant family history of neurological, ophthalmological, or neurocutaneous disorders, including seizures, cerebrovascular disease, glaucoma, or SWS.

On general examination, his temperature was 37.2 °C, heart rate was 160 beats per minute, blood pressure was 110/65 mmHg, and respiratory rate was 35 breaths per minute. His pupils were equal in size and reactive to light. His Glasgow Coma Scale (GCS) score was 8 (E1V3M4). Physical examination revealed an extensive PWS involving the right cheek and mandible. The lesion was asymmetric, more prominent on the right side of the face (Figure 1A). It had been present since birth but had been regarded by the family as an ordinary birthmark, and no medical evaluation had been sought before this admission. Cardiac auscultation revealed normal heart sounds without murmurs. Neurological examination showed right-sided hemiplegia, with reduced muscle strength (grade III) in both the right upper and lower limbs. There were no signs of meningeal irritation or positive Babinski reflexes, and both patellar and ankle reflexes were normal.

**Figure 1:**
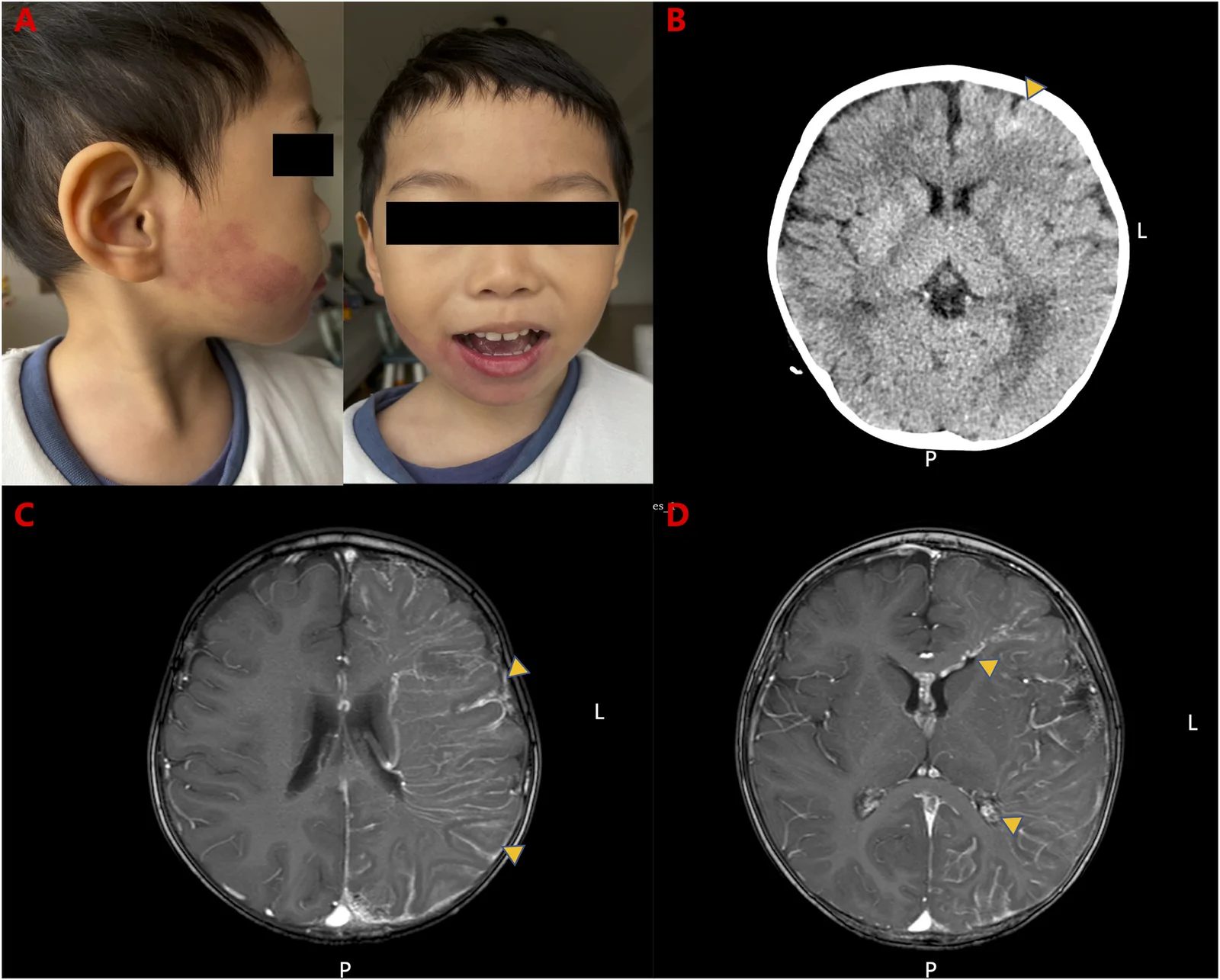
Clinical features and neuroimaging findings. **(A)** The patient had an extensive port-wine stain involving the right cheek and mandible. **(B)** Non-contrast head CT obtained at admission showing a small hyperdense hematoma in the left frontal lobe (arrow). **(C,D)** Axial gadolinium-enhanced T1-weighted MRI showing left-sided gyriform leptomeningeal enhancement extending into the cortical sulci and associated abnormal pial vessels (arrowheads), consistent with pial angiomatosis.

After the trauma, an initial head CT scan performed at a local hospital showed a small hematoma in the left frontal lobe. Unfortunately, the original imaging data were not available for review. On admission, head CT demonstrated a small high-density nodule in the left frontal lobe, strongly suggestive of a small hematoma (Figure 1B). Slight widening of the left frontotemporal extracerebral space was also observed. Blood tests showed a mild increase in C-reactive protein (CRP, 10.2 mg/L; reference value, 8 mg/L), white blood cell count (12,100 cells/µL; reference range, 5,100–14,100 cells/µL), and neutrophils (63.9%; reference range, 13%–55%). Routine liver and kidney function tests, electrolyte levels, and the coagulation profile were normal. In view of the IH and neurological manifestations, intravenous midazolam was administered, which relieved the seizures and restored consciousness. Intravenous glycerol and hypertonic saline were also used to reduce intracranial pressure. After the patient's condition improved and no contraindication to lumbar puncture was identified, cerebrospinal fluid analysis was performed and showed normal results. Other potential causes of seizures were also excluded, including cardiac disease, with normal echocardiography and electrocardiography findings. However, because neurological deficits persisted and PWS was present, further evaluation for possible SWS was warranted. MRI was performed, and revealed mild atrophy in the left cerebral hemisphere. Gadolinium-enhanced MRI showed multiple gyriform enhancing linear shadows and leptomeningeal enhancement along the cortex extending into the sulci, findings indicative of pial angiomatosis characteristic of SWS (Figures 1C,D). Magnetic resonance venography (MRV) revealed multiple venous vascular networks in the left leptomeningeal distribution, together with thickening and tortuosity of the left choroid plexus. Electroencephalography (EEG) showed slow waves in the left cerebral hemisphere, consistent with the clinical presentation. After multidisciplinary consultation with neurologists, dermatologists, and radiologists, SWS was diagnosed on the basis of PWS, pial angiomatosis, and seizures. An ophthalmological consultation was obtained during hospitalization. Intraocular pressure was normal range in both eyes, and anterior-segment and fundus examinations revealed no abnormalities or evidence of glaucoma. Subsequent ophthalmological follow-up findings remained normal.

One week after admission, oral levetiracetam and valproic acid were prescribed, achieving seizure control. Three months after discharge, low-dose aspirin (4 mg/kg/day) was initiated, consistent with recommendations for children younger than 3 years who have involvement of three or more brain lobes (1). He has remained seizure-free for more than two years, with only mild right hemiparesis at the latest follow-up. Two years later, follow-up brain MRI showed no disease progression. The scan demonstrated atrophy of the left parietal, occipital, and frontal gyri, widening of the cerebral sulci, and linear leptomeningeal enhancement extending into the sulci, consistent with the previous findings.

Following counseling, the patient's parents reported that they understood the diagnosis of SWS, the treatment plan, and the need for long-term follow-up. They were satisfied with the sustained seizure control and overall clinical improvement, despite the persistence of mild right hemiparesis, and remained committed to the recommended treatment and follow-up plan. The clinical presentation, investigations, treatment, and follow-up are summarized in Table 1.

**Table 1:** Clinical timeline.

Time	Clinical event
Day −4	Two minor falls caused superficial abrasions with bleeding on the left forehead.
Day −3	Irritability, vomiting, and gait instability developed, followed by lethargy.
Day −2	Recurrent right focal motor seizures progressed to status epilepticus.
Day 0	Emergency presentation with GCS 8 and right hemiplegia; CT showed a small left frontal hematoma.
Hospitalization	Midazolam controlled seizures; intracranial-pressure treatment was given. Contrast-enhanced MRI and EEG supported the diagnosis of SWS.
Day +7	Oral levetiracetam and valproic acid were started, with seizure control.
Month +3	Low-dose aspirin (4 mg/kg/day) was initiated after discharge.
Year +2	The child remained seizure-free with mild right hemiparesis; follow-up MRI showed no disease progression.

GCS, Glasgow Coma Scale; CT, computed tomography; MRI, magnetic resonance imaging; EEG, electroencephalography.

## 3 Discussion

SWS is a rare neurocutaneous disorder characterized by dilated post-capillary venules involving the skin, eye, and leptomeninges of the brain. Early signs, particularly PWS, may be subtle or misinterpreted, delaying diagnosis until neurological or ocular complications, such as seizures or glaucoma, or other complications emerge (3, 4). Brain involvement commonly presents with seizures, stroke-like episodes, hemiparesis, cerebral atrophy and calcifications, migraine headaches, and intellectual disability (1). By contrast, IH is an uncommon complication of SWS, with the available evidence derived mainly from isolated case reports (Table 2) (7–15). Bleeding has been reported in subdural, lobar, thalamic, and intraventricular locations. Among adults, reported presentations have included seizures, hemiplegia, and headache (8, 10, 13–15). In pediatric cases, common manifestations have included hemiplegia and signs of increased intracranial pressure, such as vomiting, irritability, impaired consciousness, and coma (7, 9, 11, 12).

**Table 2:** Summary of Sturge–Weber syndrome with intracranial hemorrhage.

Author/year	Age	Gender	SWS type	Symptoms before hemorrhage	Location of hemorrhage	Symptoms with hemorrhage onset	Therapy	Outcome
Lopez, 2013 (7)	20m	Male	I	Epilepsy, left spastic hemiplegia, and bilateral upper facial port-wine stain	Subdural hematoma	Drowsiness and temporary mild left-sided weakness worsening	Phenobarbital, aspirin, and Rehydrated	Seizure-free, mild motor delays remain
Nakajima et al., 2014 (9)	2y10m	Female	I	Epilepsy and left-sided facial port-wine stain	Right thalamic hemorrhage with ventricular spread	Deep coma and left hemiplegia	Phenobarbital, emergency ventricular drainage	Recovered motor function; 2-year follow-up: severe psychomotor delay.
Pinzon, 2024 (13)	41y	Male	NA	No previous neurological symptoms; uncontrolled hypertension and smoking	Right thalamus	Left hemiparesis and focal seizures	Conservative management, antihypertensive therapy, and antiseizure medication	Modified Rankin Scale score of 2 at 6 months
Pozzati, 1983 (12)	9y	Male	III	Epilepsy	Left parietal lobe, ipsilateral ventricle	Headache, vomiting, right hemiparesis, and homonymous hemianopsia	Left parieto-occipital craniotomy	Seizure free, stable right hemiparesis
Salih et al., 2006 (11)	4m	Male	I	Epilepsy and flat angiomatous nevus on upper left face	Left frontal and parietal lobes	Irritability, generalized seizures, and right-sided hemiparesis	NA	Alive
Aguglia et al., 2008 (15)	37y	Female	I	Right arm weakness, left facial port-wine stain, left severe glaucoma	Left temporal lobe	Focal motor seizures, Todd's paresis	Levetiracetam, carbamazepine	Seizure free on carbamazepine
Anderson, 1974 (10)	32y	Female	I	Extensive port-wine nevus on face, neck, and chest	Subarachnoid	Severe headache and stiff neck	Diphenylhydantoin	Free of headache
Chonan, 2016 (8)	62y	Male	I	Visual impairment in the right eye, right-sided facial port-wine stain	Left thalamic	Coma and right-sided hemiparesis	Emergency ventricular drainage	Consciousness and hemiparesis improved.
Dolkart, 1995 (14)	24y	Female	I	Epilepsy, left forehead and eye port-wine stain	Left lateral intraventricular	Acute repetitive focal seizure, postictal aphasia, and homonymous hemianopsia	High doses of phenobarbital, phenytoin, lorazepam, and gabapentin.	Best controlled with phenytoin and carbamazepine.

Several previously reported pediatric patients had seizures or other neurological abnormalities before the hemorrhage (7, 9, 11, 12). In contrast, our patient had no recognized neurological history before admission. His congenital PWS had been regarded as a birthmark and had not prompted medical evaluation. The small frontal hematoma was therefore the first identified neurological manifestation, followed by status epilepticus and hemiplegia. Minor trauma preceded hemorrhage in both our patient and the 20-month-old boy reported by Lopez et al (7). However, neither case established a causal relationship. This case indicates that IH may be the first recognized neurological manifestation of previously undiagnosed SWS, even in a child without preceding seizures or developmental concerns.

One possible explanation for hemorrhage in SWS is failure of the abnormal venous drainage system. Leptomeningeal vascular malformations impair superficial cortical venous outflow, redirecting blood through dilated deep medullary and subependymal veins. This altered circulation can lead to venous congestion, reduced perfusion, and limited hemodynamic reserve (1, 16). If collateral drainage is inadequate or venous outflow becomes acutely obstructed, the resulting increase in venous pressure may predispose to hemorrhage. This mechanism is supported by two reported cases of thalamic hemorrhage in which MRV showed occlusion or severe narrowing of major dural sinuses and internal cerebral veins (8, 9). In another adult case, CT angiography identified a small angiomatous nidus draining into an ectatic venous collector adjacent to the hematoma (15). In our patient, MRV showed an abnormal left leptomeningeal venous network, together with thickening and tortuosity of the left choroid plexus, supporting the presence of abnormal venous drainage. However, the available imaging did not demonstrate definite acute venous thrombosis. The frontal hematoma may therefore have arisen within a vulnerable venous circulation, although its precise source remains uncertain.

The contribution of trauma is also uncertain. A toddler with known SWS developed a subdural hematoma after a mechanical fall, whereas transient hemiplegia after minor head injury has been reported without hemorrhage (7, 17). In our patient, two falls preceded clinical deterioration, and the hematoma was detected shortly afterward. This temporal relationship raises the possibility that trauma precipitated bleeding in a brain with limited venous reserve. However, the injuries were minor, no pre-trauma imaging was available, and the falls could themselves have represented early neurological dysfunction. Minor trauma should therefore be regarded as a possible precipitating factor and should not preclude evaluation for SWS-related cerebrovascular abnormalities.

Neuroimaging is important to defining the extent of brain involvement and venous abnormalities in SWS. Current consensus recommends contrast-enhanced MRI after the onset of neurological symptoms and selective imaging in asymptomatic infants with extensive high-risk PWS (18). However, the optimal timing of neuroimaging evaluations in patients with suspected SWS remains uncertain (19, 20). Luat et al. suggested that MRI should be performed after 1 year of age (20). Indirect MRI signs in infants with SWS, such as the choroid plexus size ratio and thickness, have shown high sensitivity and specificity for diagnosis (21). Early neuroimaging, potentially before neurological symptoms emerge, may help detect brain involvement and inform longitudinal assessment, particularly in patients with PWS involving the hemiface, forehead, or medial face (5, 22).

Low-dose aspirin is used empirically in SWS to limit platelet-mediated thrombosis associated with impaired venous outflow. By reducing venous stasis and secondary cerebral hypoperfusion, it may decrease stroke-like episodes and possibly seizures (1, 23, 24). Retrospective cohorts and small presymptomatic series suggest potential neurological benefits, but no randomized trial has established its efficacy (7, 25, 26). Most reported adverse effects were minor, although clinically significant bleeding, including subdural hematoma after minor trauma, has been reported (23).

Evidence regarding aspirin use after IH in patients with SWS is limited. Current guidelines for adults suggest that, when a clear indication exists, antiplatelet therapy may be resumed after spontaneous intracerebral hemorrhage if the expected vascular benefit outweighs the risk of recurrent bleeding (27). In our patient, aspirin was initiated three months after discharge. Extensive brain involvement and abnormal venous drainage raised concern for persistent venous stasis, thrombosis, and secondary hypoperfusion. The potential benefit of limiting further thrombotic-ischemic injury and stroke-like episodes was weighed against the risk of recurrent hemorrhage. No recurrent hemorrhage occurred during more than two years of follow-up. Nevertheless, this observation cannot establish safety. More clinical experience is needed to determine when aspirin can be used safely after IH in patients with SWS and which patients are most likely to benefit.

## 4 Conclusion

This study describes that IH may be the initial presentation of SWS in a child without a known neurological history. Timely neuroimaging and careful assessment of cerebral venous abnormalities may facilitate diagnosis and inform individualized management. More reports of cases and longer follow-up are needed to understand why IH occurs, what outcomes can be expected, and how best to manage affected children.

## Data Availability

The original contributions presented in the study are included in the article/Supplementary Material, further inquiries can be directed to the corresponding authors.
